# Macropore Regulation of Hydroxyapatite Osteoinduction via Microfluidic Pathway

**DOI:** 10.3390/ijms231911459

**Published:** 2022-09-28

**Authors:** Feng Shi, Xin Fang, Teng Zhou, Xu Huang, Ke Duan, Jianxin Wang, Shuxin Qu, Wei Zhi, Jie Weng

**Affiliations:** 1 Key Laboratory of Advanced Technologies of Materials (Ministry of Education), School of Materials Science and Engineering, Southwest Jiaotong University, Chengdu 610031, China; 2 Collaboration and Innovation Center of Tissue Repair Material Engineering Technology, College of Life Science, China West Normal University, Nanchong 637009, China

**Keywords:** calcium phosphate ceramics, macropore structure, osteoinduction, microfluidic pathway, finite element analysis

## Abstract

Macroporous characteristics have been shown to play a key role in the osteoinductivity of hydroxyapatite ceramics, but the physics underlying the new bone formation and distribution in such scaffolds still remain elusive. The work here has emphasized the osteoinductive capacity of porous hydroxyapatite scaffolds containing different macroporous sizes (200–400 μm, 1200–1500 μm) and geometries (star shape, spherical shape). The assumption is that both the size and shape of a macropore structure may affect the microfluidic pathways in the scaffolds, which results in the different bone formations and distribution. Herein, a mathematical model and an animal experiment were proposed to support this hypothesis. The results showed that the porous scaffolds with the spherical macropores and large pore sizes (1200–1500 μm) had higher new bone production and more uniform new bone distribution than others. A finite element analysis suggested that the macropore shape affected the distribution of the medium–high velocity flow field, while the macropore size effected microfluid speed and the value of the shear stress in the scaffolds. Additionally, the result of scaffolds implanted into the dorsal muscle having a higher new bone mass than the abdominal cavity suggested that the mechanical load of the host tissue could play a key role in the microfluidic pathway mechanism. All these findings suggested that the osteoinduction of these scaffolds depends on both the microfluid velocity and shear stress generated by the macropore size and shape. This study, therefore, provides new insights into the inherent osteoinductive mechanisms of bioceramics, and may offer clues toward a rational design of bioceramic scaffolds with improved osteoinductivity.

## 1. Introduction

Osteoinductive biomaterials are promising bone grafting materials in orthopedic clinics. Among these materials, calcium phosphate (CaP) bioceramics have received extensive interest because of their chemical similarity to bone minerals [1,2,3,4,5,6,7]. In addition to chemical composition, the macro-pore structure is another important factor in the bone-inducing potential of biomaterials [8]. Pore penetration and macro-pore morphology, such as pore size [9,10], geometry [11,12], orientation, porosity, interconnected pore size, and so on, determine the growth of the blood vessels [8,13,14], and the distribution of nutrients, oxygen, cells and tissues in the scaffolds, as well as the total surface area available for material–cell interactions [15,16,17,18]. Earlier studies have found that porous materials with a pore size of ≥400 μm and the interconnective pore diameter of ≥150 μm, provide an ideal space for bone tissue growth [19,20,21]. However, the mechanisms underlying bone formation and distribution in such scaffolds remain elusive, and guidelines for the optimization of products are lacking.

In vitro bioreactor studies found that the microfluid dynamic environment (mainly including microfluid velocity and fluid shear stress), formed in scaffolds after culture medium perfusion, has an important effect on the cells’ distribution, growth, and differentiation on these scaffolds [22,23,24]. The main factors affecting the microfluid dynamic environment include the perfusion velocity of the fluid medium to the scaffolds and the pore structure of the scaffolds [25,26]. That means that when scaffolds are implanted in vivo, due to the impact of the stress load of implantation sites, body fluid as a fluid medium will form a microfluid dynamic environment in the scaffolds. Therefore, the macro-pore structure properties of the materials are transformed into a biological response regulating signals, and play a role in the osteogenesis. However, due to the lack of suitable micro-sensors, the local microfluid velocity and fluid shear force in the scaffolds cannot be measured [25,27,28]. In this case, it is feasible to study the correlation between the material pore structure, the microfluid environment and the bone formation, with computational fluid dynamics (CFD), and finally establish an accurate quantitative relationship.

In this study, two kinds of hydroxyapatite (HA) scaffolds, hydroxyapatite spheres accumulating scaffolds (HASAs) and hydroxyapatite porogen-pore scaffolds (HAPPs), with different macro-pore shapes and size distributions were fabricated with the sol-gel method. The two kinds of scaffolds had star-shaped pores and spherical pores, respectively, good interconnectivity and similar microstructures. After the two kinds of scaffolds were characterized, they were implanted in the dorsal muscle and abdominal cavities of adult dogs, to compare the effects of different macro-pore shapes and/or different macro-pore sizes on the ectopic bone formation ability of the scaffolds. After implantation for 1 month, 3 months and 6 months, the scaffolds were analyzed for histology, histomorphometry and biomechanics. Finally, mathematical models for 3D scaffolds of the HASAs and HAPPS were developed, and a finite element analysis (FEA) was analyzed for the microfluid dynamic environment using computational fluid dynamics (CFD). The results confirmed the hypothesis that both the size and shape of the macropores affected the microfluidic pathways in the scaffolds, and these may underlie the bone formation and distribution in these scaffolds. The goal was to better understand the role of microfluidic environments in regulating the osteoinduction of macroporous CaP ceramics.

## 2. Results

### 2.1. The Macro-Pore Structure Properties of HASAs and HAPPs

**Figure 1:**
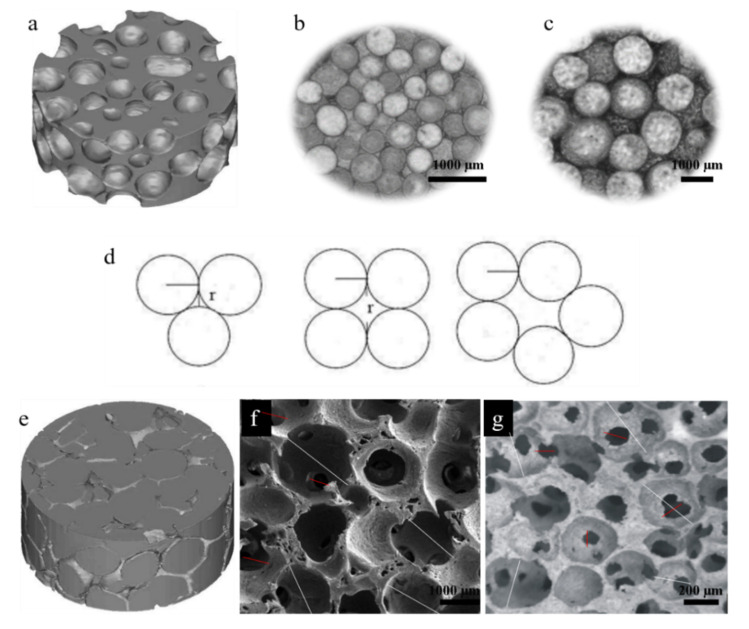
3D reconstruction using Micro-CT and showing macropore of HASAs and HAPPs.The stellate-shape macropore of HASAs (**a**). The pore size was decided by the size of HA spheres ((**b**): S-HASAs; (**c**): L-HASAs) and the arrangement of the spheres (**d**). The spherical-shape macropore of HAPPs (**e**). SEM showed the macropores and interconnective pores of L-HAPPs (**f**) and S-HAPPs (**g**).

HASAs and HAPPs are porous cylinders with a height of 2–2.5 cm and a diameter of 1.5 cm. The macro-pore structures of all the scaffolds were prepared and characterized as reported previously [29]. The HAPPs had higher total porosity and macro-porosity than the HASAs, while the two kinds of scaffolds had similar micro-porosities. Micro-CT reconstruction found the macro-pore shapes of the HASAs to be stellate-shaped, which were formed by the HA spheres accumulating (Figure 1a). That is to say, the macro-pore structure properties of the HASAs were determined by the size of the HA spheres and the arrangement of the adjacent spheres. The arrangements of the S-HASAs (Figure 1b) and L-HASAs (Figure 1c) were depicted by three-ball accumulations, four-ball accumulations and five-ball accumulations (Figure 1d). The minimum macro-pore size existed in the three-ball accumulations, and the maximum macro-pore size was smaller than the diameter of the HA sphere. The micro-CT reconstruction showed the macro-pore shapes of the HAPPs were spherical-shaped (Figure 1f). Each macro-pore had two or more inter-connective pores. The ratio of the sizes of inter-connective pore/macro-pores was ~0.4 (Figure 1g,h). The porous structures of all kinds of scaffolds with excellent inter-connectivity were beneficial to humoral flow, protein adhesion, cell migration and tissue ingrowth.

### 2.2. General Conditions of Animals

Within 3 days after the operation, the animals resumed activities and normal water/food intake. The surgical wounds healed normally. Three weeks after the operation, there was no swelling, inflammation, nor tissue necrosis around the incisions.

### 2.3. The Histological Characterization of Ectopic Bone Formation and Blood Vessel Ingrowth

**Figure 2:**
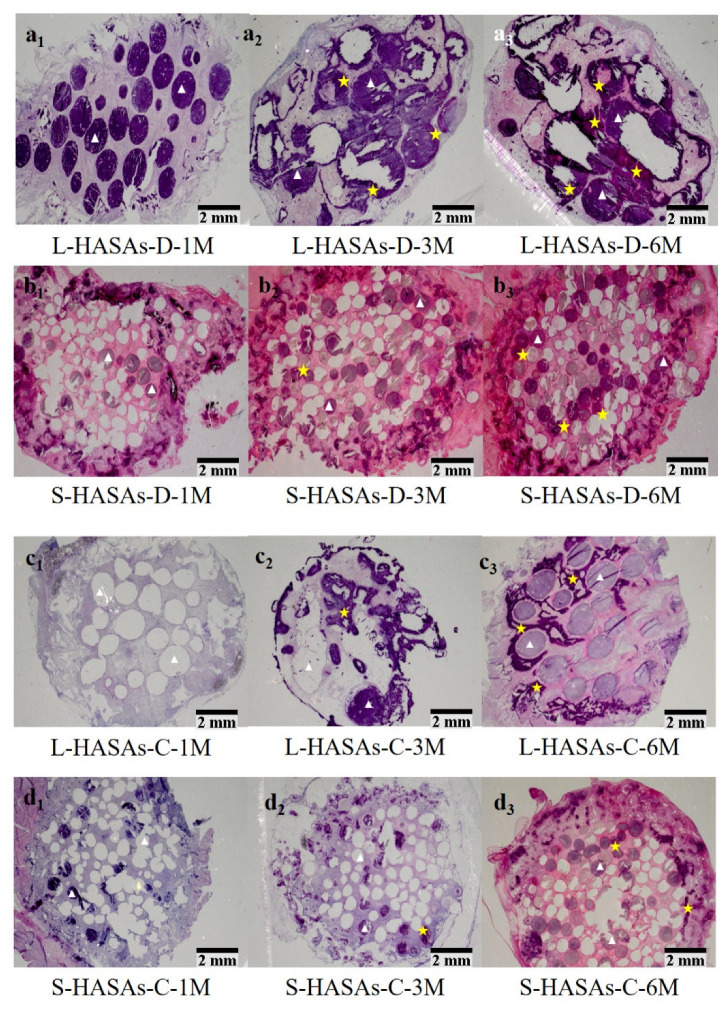
Relationship between ectopic bone formations of HASAs and the macropore sizes implanted in dorsal muscle/abdominal cavity by H&E staining. (**a_1_**–**a_3_**) L-HASAs implanted 1, 3 and 6 months, (**b_1_**–**b_3_**) S-HASAs implanted 1, 3 and 6 months, (**c_1_**–**c_3_**) L-HASAs implanted 1, 3 and 6 months, (**d_1_**–**d_3_**) S-HASAs implanted 1, 3 and 6 months. With the implantation time extended, new bone tissues were found in all scaffolds, which was better in the L-HASAs than those in the S-HASAs. White triangles show HA materials, yellow stars show new bone. The bar = 2 mm.

**Figure 3:**
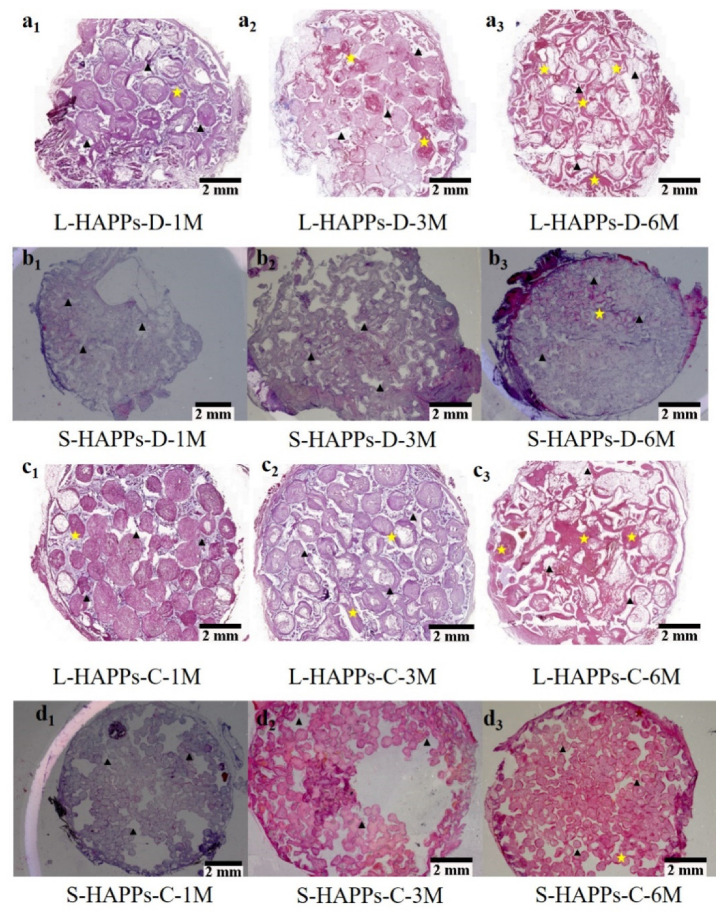
Relationship between the ectopic bone formations of HAPPs and the macropore sizes implanted in dorsal muscle/abdominal cavity by H&E staining. (**a_1_**–**a_3_**) L-HAPPs implanted 1, 3 and 6 months, (**b_1_**–**b_3_**) S-HASAs implanted 1, 3 and 6 months, (**c_1_**–**c_3_**) L-HAPPs implanted 1, 3 and 6 months, (**d_1_**–**d_3_**) S-HASAs implanted 1, 3 and 6 months. With the implantation time extended, new bone tissues were found in all scaffolds, which was better in the L-HAPPs than those in the S-HAPPs. Black triangles show HA materials, yellow stars show new bone. The bar = 2 mm.

The hematoxylin and eosin (H&E) staining results (Figure 2 and Figure 3) showed the ectopic bone formation and distribution in the scaffolds. One month after implantation, no new bone tissue was observed in the L-HASAs, S-HASAs and S-HAPPs, neither in the dorsal muscle nor in the abdominal cavity (Figure 2a_1_,b_1_ and Figure 3a_1_,b_1_). However, primary callus was widely distributed in the central and outer areas of the L-HAPPs (Figure 2c_1_ and Figure 3c_1_). Even a small amount of new bone tissue appeared in the central area of the L-HAPPs. Three months after implantation, new bone tissue was seen in the outer area of the L-HASAs with a colony and diffused distribution both in dorsal muscles and in the abdominal cavities (Figure 2a_2_ and Figure 3a_2_), while new bone tissues were only sporadically present in the outer areas of the S-HASAs (Figure 2b_2_ and Figure 3b_2_). New bone tissue was also evident in the central and outer areas of the L-HAPPs (Figure 2c_2_ and Figure 3c_2_), while only a small amount of callus was observed in the central and outer areas of the S-HAPPs (Figure 2d_2_ and Figure 3d_2_). Six months after implantation, more new bone tissues gradually grew into the central area of the L-HASAs, showing an increased bone maturation and the appearance of a medullary cavity (Figure 2a_3_ and Figure 3a_3_). There was a similar trend of new bone formation in the S-HASAs, but with fewer bone tissues and an absence of a medullary cavity (Figure 2b_3_ and Figure 3b_3_). Meanwhile, new bone tissues with medullary cavities widely appeared in the central and outer areas of the L-HAPPs (Figure 2c_3_ and Figure 3c_3_), whereas a small amount of new bone tissues was observed in the S-HAPPs (Figure 2d_3_ and Figure 3d_3_). In comparison, at each time point, the scaffolds implanted in the abdominal cavities exhibited less and later bone formation (vs. in dorsal muscles).

**Figure 4:**
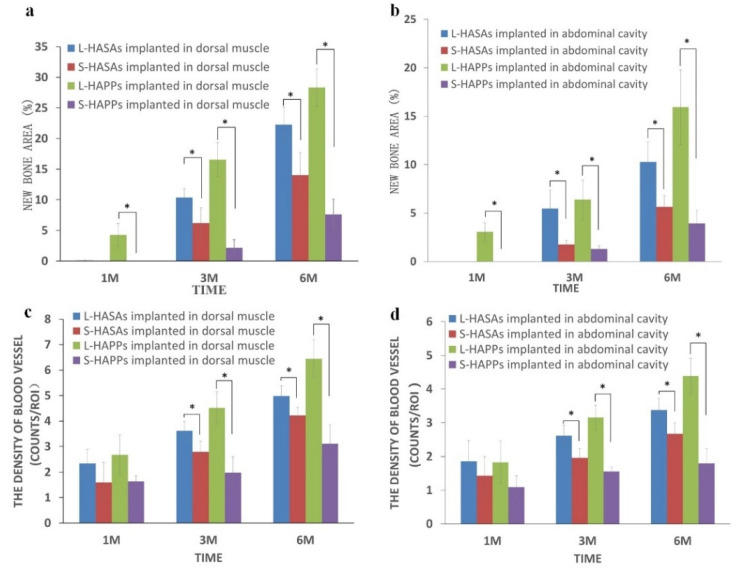
New bone formation and vessel density in HA porous scaffolds with different macropore sizes implanted in dorsal muscle and abdominal cavity using histomorphometry analysis. (**a**): new bone formation area % of HA scaffolds implanted in dorsal muscle; (**b**): new bone formation area % of HA scaffolds implanted in abdominal cavity; (**c**): vessel density of HA scaffolds implanted in dorsal muscle; (**d**): vessel density of HA scaffolds implanted in abdominal cavity (* *p* < 0.05).

A histomorphometric measurement (Figure 4a,b) found that in the dorsal muscle implantations, regardless of the kind of scaffolds, there was more active new bone formation and more new bone tissue quantity in the L-HASAs and the L-HAPPs than those in the S-HASAs and the S-HAPPs. Interestingly, the new bone formation and new bone tissue quantities of the L-HAPPs were significantly higher than those of the L-HASAs, while those of the S-HAPPs were significantly lower than those of the S-HASAs from 1 month to 6 months’ implantation. In addition, a similar trend was observed in the abdominal cavity implantations.

One month after implantation, new bone formation was only observed in the L-HAPPs, both in the dorsal muscle (4.27 ± 1.85%) and in the abdominal cavity (3.06 ± 0.94%). Six months after implantation, the ratio of new bone formation in the L-HAPPs was 28.31 ± 3.06% in the dorsal muscle and 15.93 ± 3.87% in the abdominal cavity. In comparison, for the L-HASAs, new bone formation was 22.27 ± 2.89% in the dorsal muscle and 10.28 ± 2.06% in the abdominal cavity (Figure 4a,b). For the S-HAPPs, it was 7.61 ± 2.48% in the dorsal muscle and 3.94 ± 1.33% in the abdominal cavity. For the S-HASAs, it was 14.0 ± 3.67% in the dorsal muscle and 5.65 ± 1.17% in the abdominal cavity (Figure 4a,b). Throughout this study, the S-HAPPs exhibited inferior osteoinduction to the other three groups.

The measured blood vessel density in the groups at the early implantation stage showed no significant difference for vascularization of all the scaffolds. Subsequently, the new vessel densities in the four groups followed similar trends to the new bone formations (Figure 4c,d). The amount of vessels/mm^2^ in the L-HAPPs group (6.44 ± 0.75 mm^−2^ in the dorsal muscle and 4.38 ± 0.53 mm^−2^ in the abdominal cavity) was significantly higher than the L-HASAs group (4.98 ± 0.4 mm^−2^ in the dorsal muscle and 3.17 ± 0.51 mm^−2^ in the abdominal cavity) at 6 months’ implantation. Six months after implantation, the density in the S-HASAs group (4.22 ± 0.37 mm^−2^ in the dorsal muscle and 2.87 ± 0.39 mm^−2^ in the abdominal cavity) was significantly higher than that in the S-HAPPs (3.11 ± 0.74 mm^−2^ in the dorsal muscle and 1.9 ± 0.63 mm^−2^ in the abdominal cavity).

These results showed that new bone and vessel formation was better in the L-HAPPs and L-HASAs than those in the S-HAPPs and S-HASAs, as the implantation time extended. In summary (Table 1), the new bone formation in the L-HASAs and L-HAPPs after being implanted in the dorsal muscle and the abdominal cavity were better than those of the S-HASAs and S-HAPPs, respectively. The scaffolds implanted in the abdominal cavities experienced less and later bone formation at each time point than those in the dorsal muscles. Moreover, the new bone distribution in the HASAs mainly appeared in the outer area of the scaffolds, while those in HAPPs mostly existed both in the central and outer areas of the scaffolds since the early stage.

### 2.4. Compressive Strength of Reconstructed Scaffolds

**Figure 5:**
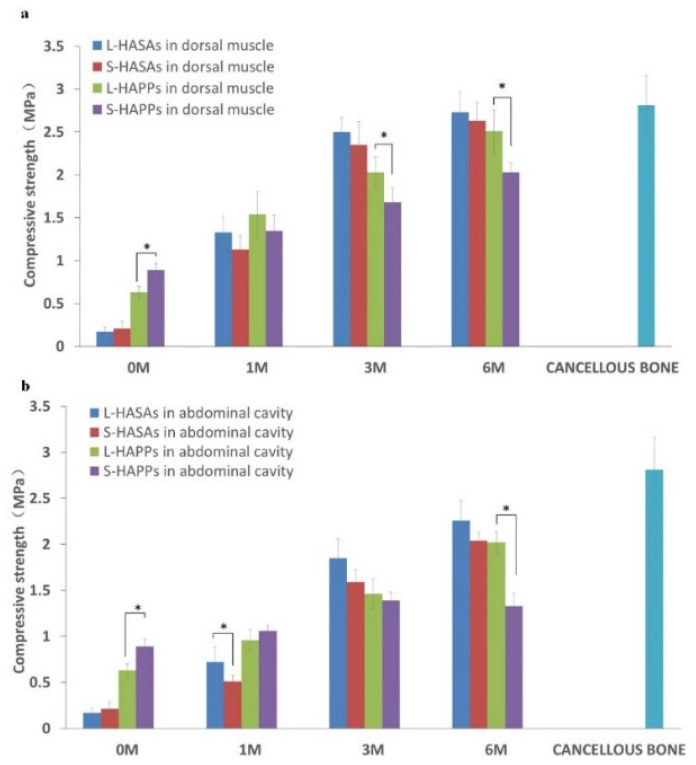
The compressive strength in HA porous scaffolds with different macropore sizes implanted in dorsal muscle (**a**) and abdominal cavity (**b**) (* *p* < 0.05).

Figure 5 showed L-HASAs and S-HASAs had a similar strength before implantation, while the L-HAPPs had a significantly lower compressive strength than the S-HAPPs. Following the ingrowth and filling of tissue, and the increase in new bone formation in the scaffolds, the compressive strengths of all scaffolds increased at different degrees. The L-HASAs and L-HAPPs increased more prominently. Six months after implantation, the compressive strength of the L-HASAs implanted in the dorsal muscle and the abdominal cavity increased approximately 16-fold (from 0.17 MPa to 2.73 MPa) and 13.3-fold (from 0.17 MPa to 2.26 MPa), respectively. In comparison, the L-HAPPs implanted in the dorsal muscle and the abdominal cavity increased almost 4-fold (from 0.63 MPa to 2.51 MPa) and 3.2-fold (from 0.63 MPa to 2.02 MPa), respectively. The S-HASAs increased almost 12.5-fold (from 0.21 MPa to 2.63 MPa) and 3.2-fold (from 0.21 MPa to 2.04 MPa), respectively. The S-HAPPs increased almost 2.3-fold (from 0.89 MPa to 2.03 MPa) and 1.5-fold (from 0.89 MPa to 1.33 MPa), respectively. Compared with the other three groups, the S-HAPPs group was obviously lower in compressive strength because of the lesser amount and maturity of new bone formations at 6 months.

Additionally, the results showed that the compressive strengths of the scaffolds implanted in the dorsal muscle at three time points were higher than of those implanted in the abdominal cavity, which were consistent with the histomorphometric findings, and indirectly showed that the new bone formation in the dorsal muscle was better than in the abdominal cavity.

### 2.5. Numerical Simulation of Fluid Flow in the Scaffolds

**Figure 6:**
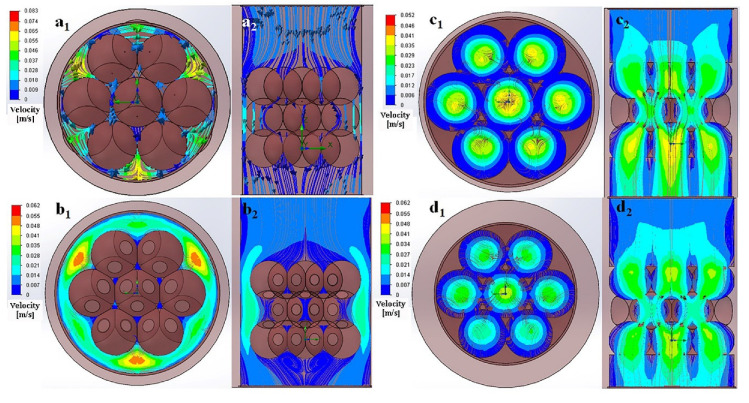
CFD fluid simulation analysis of HASAs and HAPPS scaffolds reveal the relationship between distribution of flow field and macropore size. The distribution of flow field on the horizontal plane in: L-HASAs (**a_1_**), S-HASAs (**b_1_**), L-HAPPs (**c_1_**), S-HAPPs (**d_1_**). The distribution of flow field on the sagittal plane in: L-HASAs (**a_2_**), S-HASAs (**b_2_**), L-HAPPs (**c_2_**), S-HAPPs (**d_2_**). Color bar shows flow velocity.

A CFD simulation showed that the flow through the scaffold’s microarchitectures was highly non-uniform (Figure 6), the highest flow speeds in the HASAs (star-shape macro-pores) appeared near the edges (red area of Figure 6a_1_,b_1_), while the lowest speeds were at the central region (blue area of Figure 6a_1_,b_1_). The distribution of local shear stresses on the whole fluid pathway surfaces of the HASAs is shown in Figure 6a_2_,b_2_. The highest values for local shear stresses were found on the walls of pores close to the edge of the scaffold (red area of Figure 6a_2_,b_2_). In the L-HASAs, the highest surface shear stress in the range from ≈ 1.02 to 1.15 Pa correlated to the highest flow speed from ≈ 0.039 to 0.044 m/s; in the S-HASAs, the highest surface shear stress in the range from ≈ 4.50 to 5.07 Pa correlated to the highest flow speed from ≈ 0.053 to 0.059 m/s. For the HAPPs (sphere-shaped macro-pores), the highest flow speeds were found in the middle of the pores in the central region (yellow or green area of Figure 6c_1_,d_1_), while the lowest speeds occurred near the surface of the pores (blue area of Figure 6c_1_,d_1_). The flow speeds were also high near the interconnection of the pores. The distribution of local shear stresses on the whole fluid pathway surfaces of the HASAs is shown in Figure 6c_2_,d_2_. The highest values for the local shear stresses were found on the surface of the interconnection of the pores (red area of Figure 6c_2_,d_2_). In the L-HAPPs, a surface shear stress around an interconnected pore in the range from ≈1.80 to 0.90 Pa correlated to a flow speed from ≈0.025 to 0.034 m/s; in the S-HAPPs, a surface shear stress around an interconnected pore in the range from ≈3.91 to 5.87 Pa correlated to a flow speed from ≈0.038 to 0.044 m/s.

The results suggest that the macro-pore shape of the scaffold affects the distribution of the fluid field and fluid shear stress, while the macro-pore size of the scaffold affects the flow speed and the value of shear stress.

## 3. Discussion

In this study, four kinds of porous HA scaffolds with the same chemical phase composition and micro/nano structures [29,30,31], but different macro-pore structures (size, shape) were used to ensure the material variables caused by the macro-pore structural variables. The HASAs and HAPPs had two sizes of macro-pore, 0.2–0.4 mm and 1.2–1.5 mm, respectively, which can be seen as two ranges of macro-pore size, small size and large size. Additionally, other studies have reported that the macro-pores of an HA porous scaffold with inter-connectivity network pores with the size of 140–160 μm could be completely filled by bone tissue after implantation in the orthotopic bone tissue [32,33,34,35]. Therefore, the HAPPs used in this study were selected to an inter-connective pore/macro-pore size ratio of ~0.4, to avoid the effect of the size of inter-connective pores.

The results of the histological analysis indicated that the pore structure of the HA porous scaffolds was more beneficial for the growth of cells and tissues, and the formation of new bone, when the size of the macro-pores were >400 μm (Figure 2 and Figure 3). Previous research has found that porous α-TCP bone cement columns with the 150 μm macro-pore size had no new bone formation after 90–180 days’ subcutaneous implantation in goats, and lost the integrity of the scaffolds after 3 months’ implantation [16]. Hence, the small macro-pore size was considered not conducive to new bone formation, and it was believed that the loss of the macro-pore structure of scaffolds would impede the transport of nutrition in the scaffolds, and be not conducive to protein adsorption, cell adhesion and differentiation [16,36]. Although all of the kinds of HA scaffolds had good connectivity and high porosity, the osteoinduction of the L-HASAs and L-HAPPs was obviously superior to the S-HASAs and S-HAPPs. This may be due to the fact that the scaffolds with macro-pore sizes of <400 μm, especially for the HAPPs, are likely to allow the blood or tissue fluid generated during implantation to be trapped in the macro-pores, resulting in a large number of cells and proteins blocking the inter-connective pores of the scaffold, thereby reducing scaffold connectivity. This is not conducive for the vascular network of host tissue to grow into the scaffolds and to provide more adequate oxygen and nutritional support for the cells and tissues grown into the scaffold. This indicates that there may be a threshold for the macro-pore size of the HA porous scaffold to determine osteoinduction. Only when the size of a macro-pore exceeds the threshold, the scaffold would have a strong osteogenic potential.

In this study, we found that the shape of the macro-pores affected the distribution of new bone tissue in the HA porous scaffolds. The bone formation in the HASAs first appeared in the outer region of the scaffolds, and gradually grew into the central region as implantation time was extended. The bone formation in the HAPPs appeared in the central and outer regions simultaneously, and was particularly active in the central region. A previous study has reported that bone formation was observed in the concave of the graft, and had not appeared in the protruding part of the graft when rod- and disc-shaped HA scaffolds with concave surfaces were implanted into the muscle of a baboon [37]. Other studies have reported that the bone formation of porous ceramic scaffolds mainly occurred in the pore’s inner wall, near the opening of the pore after an ectopic implantation in vivo [38,39,40]. Recently, Zhang et al. has investigated the osteogenic properties of triply periodic minimum surfaces structure-based HA scaffolds, and also found that the amount of new bone formation and the distribution area above the scaffolds were significantly different from those of the HA scaffolds with cross-hatch structures [41]. Those results have indicated the influence of the shape of the macro-pores on the osteoinduction of materials [37,42]. The porous gradient distribution was more conducive to osteogenesis and maintained better mechanical properties than the uniform distribution [43]. These data should be related to the macro-pore structure affecting the distribution of blood or liquid fluid in the scaffolds, thus affecting the protein and cell adhesion, nutrient and oxygen transport.

The results of this study have also further revealed that the shape and the size of macro-pores play a synergistic effect, affecting the growth of new bone in the scaffolds. When the size is about 1300–1500 μm, spherical macro-pores are likely to be more conducive to osteogenesis, but when the size is <400 μm, stellate macro-pores may be more conducive to osteogenesis. The reason for this phenomenon may be that there exists a threshold of macro-pore size for the concave curved surface of the spherical pore structure of the HAPPs. When the pore size is below this threshold, the oxygen supply, nutrients diffusion, cell migration, and tissue growth will be hindered after a scaffold implantation. In contrast, the convex curved surface of the stellate pore structure of the HASAs was more conducive to liquid flow, protein adhesion and cell migration than the HAPPs when the size of the macro-pore was <400 μm. However, when the pore size exceeded the threshold, the spherical pore structure was more conducive to tissue growth and new bone growth, as the HAPPs have a greater interconnection of space. Hence, the L-HAPPs had better osteogenic ability than the L-HASAs, while the S-HASAs had better osteogenic ability than the S-HAPPs.

In addition, the stress environments of the implanted sites in vivo also significantly affected and regulated the process of bone formation. Under a higher stress in the dorsal muscle implantation, compared with those in the abdominal cavity, the scaffolds gradually changed to spindle-like shapes, and the new bone formation was significantly higher than those implanted in the abdominal cavity. Compared with the abdominal cavity, muscles are more commonly selected in osteoinduction research. The muscle tissue has adequate blood supply, rich interstitial cells, and a compressive stress environment following a rhythmic contraction–diastole, which is conducive to cell, oxygen and nutrients, followed by tissue fluid or granulation tissue into the scaffolds. The pressure loading on the scaffold can accelerate the degradation of the calcium phosphate ceramic material, thus forming a locally high concentration of calcium and phosphorus ions in the environment around the scaffold. Calcium and phosphorus ions formed a mineralized layer on the surface of the macro-pore wall of the scaffold, which provided a favorable environment for osteogenesis.

The biomechanical results reflected the regulatory effect of the macro-pore structure of the HA scaffolds and the stress load of the implantation sites on the new bone formation and tissue ingrowth. Three months after implantation, the compressive strength of the L-HAPPs and L-HASAs became significantly higher than that of the S-HAPPs and S-HASAs, respectively. Three and six months after implantation, tissue ingrowth and new bone formation were observed in the L-HASAs and L-HAPPs. For the L-HASAs, the HA spheres were encapsulated by new bone tissue and connective tissue as a whole, and the movement between the HA spheres was restricted, which further enhanced the compressive strength of the graft. For the L-HAPPs, excellent tissue ingrowth and wide reconstruction in the macro-pores’ walls increased the compressive strength significantly. On the other hand, since the callus formation was present in the L-HAPPs at the early implantation stage, the compressive strength of the L-HAPPs was significantly higher than that of the L-HASAs after 1 month of implantation. With the implantation time increased, the compressive strength of the S-HASAs was promoted by new bone formation, mainly in the outer region of the graft and connective tissue ingrowth. The compressive strength of the S-HAPPs was increased only moderately because of the limited bone formation and the connective tissue ingrowth. Hence, the compressive strength of the S-HASAs was substantially higher than that of the S-HAPPs 3 months after implantation. In addition, the compressive strength of the scaffolds in the dorsal muscle was significantly higher than that in the abdominal cavity at each implanted time point. The stress load of the scaffold implanted into the dorsal muscle was higher than that of the abdominal cavity, and the scaffold was located in the internal environment rich in blood supply and mesenchymal cells. Therefore, the new bone formation and tissue ingrowth of grafts implanted in the dorsal muscle were better than those implanted in the abdominal cavity. The change of the mechanical properties after the implantation of the scaffold has an important impact on its application. Compared with incorporated composite gels, which are commonly used as bone tissue engineering scaffolds to fill bone defects in non-load-bearing sites, calcium phosphate bioceramics have the potential to be applied in the repairment of load-bearing bone defects [44,45]. The important influence of the shape and size of the macropores on the change of the mechanical strength in vivo undoubtedly makes it a key consideration in the optimization of the design of the scaffolds.

The difference of new bone formation and distribution between the HASAs and HAPPs was explained by the results of the flow simulation calculation. In the microfluid environment of the HASAs, the medium–high velocity flow field was mainly distributed in the outer region. The medium–high velocity of the fluid was conducive to the transmission and diffusion of oxygen and nutrients, the metabolites exchange, the cell migration, and tissue ingrowth. Therefore, in the HASAs, the new bone formation always appeared first in the outer region, and gradually grew into the central region. Furthermore, the difference of the medium–high velocity fluids suggested that there existed a better osteogenic environment in the L-HASAs than in the S-HASAs. While the medium–high velocity flow field of the HAPPs was uniformly distributed in the whole scaffold, and the highest flow velocity was located in the central region, which was beneficial to the uniform distribution of oxygen and nutrients in the scaffolds, and also beneficial for cell migration, survival and tissue ingrowth. Hence the ectopic bone formation often occurred simultaneously in the central region and the outer region of the HAPPs. At the same time, the L-HAPPs were superior to the S-HAPPS, both in the distribution of the medium–high velocity flows and the difference of flow velocities, suggesting that the L-HAPPs had a better osteogenic environment.

In this study, we found that the size and shape of the macro-pores had an obvious effect on the new bone formation and distribution in the HA scaffolds. The flow simulation results showed that this effect was most likely to work through the microfluid environment of the scaffold. That is to say, the microfluid dynamic environment acts as a transition pathway linking the materials’ macrostructure properties to signals regulating the osteoinduction of these materials. Xu et al. have reported that the fluid dynamic environment and macrostructure properties had important effects on the cell growth and distribution in the scaffolds [25]. When the perfusion of porous scaffolds with random macrostructures at a certain velocity continued, the local proliferation rates and distributions of cells on the scaffold were different [25]. Another study has shown that the distribution of the fluid dynamic environment was highly dependent on the macro-pore distribution in the scaffold, and the different macro-pore structures provided different fluid accessibility [46]. The study has also shown that the process of the cells’ differentiation in the fluid dynamic environment was more regulated by the axial perfusion of the scaffold [47]. Porter et al. have reported that the microfluid dynamic environment can enhance the transport of oxygen and nutrients, and the exclusion of metabolites, in in vitro tissue engineering constructs, but have also provided fluid stress to stimulate cell proliferation and differentiation, while the microfluid dynamic environment was determined by the macro-porosity, the size of the macro-pores and the fluid velocity [26]. These studies have suggested close correlations among the scaffold macro-porous structures, microfluid dynamic environments and cell distribution and differentiation.

Although the results of the finite element simulation can well explain the histological results in the in vivo experiment, it was hard to completely match the simulation and animal experiment. In the process of the scaffold preparation, influenced by the process parameters and preparation methods, there were some individual differences and structural imperfections in the obtained scaffolds, thus affecting the in vivo experimental results. In the simulation calculation, the macro-pore structures of the scaffolds were set to complete interconnectivity, the sizes of the macro-pores (in the HAPPs) or the sizes of the spheres (in the HASAs) were set to a uniform value, and the ratio of the inter-connective pore size to the macro-pore size was set to 0.4. However, in the actual scaffold preparation, the sizes of pores or spheres are difficult to be completely consistent, and the ratio of the inter-connective pore sizes to the macro-pore sizes was also difficult to control in an exact value. Although the parameters of the macro-pore structure tend to theoretical configuration, the difference is inevitable.

In addition, in our finite element simulation, only one velocity-inlet value was set. However, the stress loading on the scaffold implanted in the different sites in vivo is not the same, which will affect the velocity-inlet of the fluid flow across the scaffold, and hence affect the microfluid dynamic environment of the scaffold [25]. This was demonstrated in our in vivo experimental results, the new bone formation in the scaffolds implanted in the abdominal cavity with lower stress loading was generally worse than those implanted in the dorsal muscle with higher stress loading.

**Figure 7:**
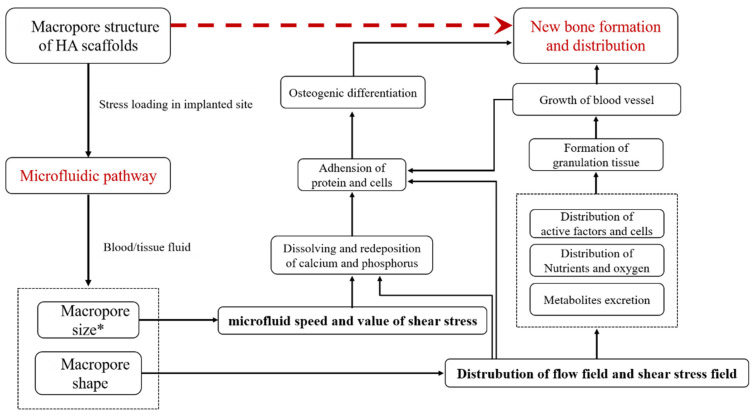
The osteoinductive mechanism of macropore structure of HA scaffolds.

Bone formation in bioceramic scaffolds is considered a dynamic equilibrium process, involving the growth of blood vessels, supersaturation of calcium ion concentrations, deposition of bioactive apatite layers, and migration, adhesion, proliferation and differentiation of cells, which are necessary for the formation of new bone [48,49,50,51,52]. On this basis, combined with the results of this study, we explained the mechanism of the formation and distribution of the new bone tissues, which were influenced by the microfluid dynamic environment (Figure 7). At the early implantation stage, blood, tissue fluid, mesenchymal cells in the surrounding tissue, and inflammatory factors were driven by the stress load of the implant site into the scaffold. In this process, the microfluid dynamic environment (fluid velocity, flow field distribution, and fluid stress distribution, etc.) within the scaffold determined the distribution of the activity factors and the cells. Subsequently, in the processes of the blood scab formation, calcium phosphate dissolution–redeposition, bone-like apatite formation, protein adhesion, granulation tissue formation and ingrowth, and the microfluid dynamic environment determined the deposition volume and distribution of the bone-like apatite and the blood vessels’ distribution. Thereafter, the mesenchymal cells recruited from the surrounding tissue and blood vessels adhered to the bone-like apatite layer, the cells proliferated and osteogenesis differentiated under chemical, surface micro-morphology and mechanical stimulation. Osteoclasts appeared, fibrous callus formed, and finally new bone appeared afterwards. The microfluid dynamic environment affected the distribution of nutrients and oxygen, the metabolites excretion, the cell adhesion and differentiation, as well as the formation and distribution of new bone in this sequence of events. Throughout these processes, the microfluid dynamic environment was determined by the size and shape of the macro-pore and the stress load at the implant site.

## 4. Materials and Methods

### 4.1. HA Scaffolds Preparation

The spherical HA powder used in this experiment was from the National Biomedical Materials Engineering Research Center of Sichuan University. Its main particle size was about 20 μm.

#### 4.1.1. Two Macro-Pore Sizes of HASAs Preparation

The HASAs were prepared by accumulating the HA spheres in porous HA tubes, using the method reported in our group [29,31,53,54]. Briefly, the HA powders were dispersed in a chitin-lithium chloride solution in dimethylformamide (all from Chengdu Kelong Chemical Co., Ltd., Chengdu, China) to form a suspension. The suspension was shaped into spheres, porous tubes, and porous discs, and subsequently sintered into ceramic objects. The sintered HA ceramic tubes and discs were reinforced by immersing in polylactide (PLA, Mw 120 K, Institute of Organic Chemistry, Chinese Academy of Sciences, Chengdu, China)/acetone solution (5% *w*/*w*). The HA spheres (diameter: 280–430 μm, 1300–1500 μm) were filled, respectively, into the porous HA tubes (external diameter: 15 mm, internal diameter: 10 mm, height: 20 mm), gently shaken to ensure the spheres were tightly packed, and finally sealed with the porous HA discs (diameter: 15 mm, height: 3 mm) to the final scaffolds (Figure 1). The HASAs with two sphere sizes were labeled the L-HASAs and the S-HASAs.

#### 4.1.2. Two Macro-Pore Sizes of HAPPs Preparation

The HAPPs were prepared by using paraffin spheres with diameters of about 3 mm and about 0.8 mm as the pore-forming agents, respectively, according to the preparation method of our earlier publication [30]. Briefly, HA powders were dispersed in a chitin-lithium chloride solution in dimethylformamide (all from Chengdu Kelong Chemical Co., Ltd., China) to form a suspension. Subsequently, the melting paraffin was dropped into a stirred polyvinyl alcohol (PVA, Chengdu Kelong Chemical Co., Ltd., China) to form the paraffin spheres. These spheres were then accumulated in a cylindrical mold and immersed in xylene (Chengdu Kelong Chemical Co., Ltd., China) and given pressure for a while to control the inter-sphere connectivity. The HA suspension was infused and gelled with water in air. Paraffin was wiped off by immersing in n-hexane (Chengdu Kelong Chemical Co., Ltd., China). The initial scaffolds were sintered into ceramic objects (Figure 2). The two macro-pore sizes of HAPPs were labeled L-HAPPs and S-HAPPs, respectively.

All the scaffolds were sealed in vacuum packages and sterilized with ethylene oxide for subsequent implantation.

### 4.2. Animal Experiment

All processes involving animal experiments were conducted in accordance with the Chinese local ethics committee and the experimental animal management system. The experiment follows the internationally recognized 3R principle (reduction, replacement, and refinement) for the more ethical use of animals, and conforms to the relevant contents of the guiding opinions on being kind to experimental animals. Twelve adult healthy dogs (1 year old, 15–20 kg) were used in this study for three time points (implantation for 1 month, 3 months, 6 months; four dogs for each time point). Immediately before surgery, sodium pentobarbital was dissolved in physiological saline (W% = 3%), sterilized by filtration, and intraperitoneally injected as a l mL/kg basis.

Two samples of four kinds of scaffolds were implanted, respectively, into the dorsal muscle and abdominal cavity of each animal, as described in detail as follows.

Dorsal muscle implantation: Scaffolds were implanted into the dorsal muscle following a previous study [55]. The dog was placed in a prone position, and the dorsal thoracolumbar area was shaved and disinfected. An incision was made along the dorsal midline from T8 to L5 to expose the para-spinal muscles. Eight intramuscular pockets (four/side) were created in the dorsal muscles parallel to the spine by blunt dissection, and a scaffold was placed in each pocket. Finally, the wound was closed in layers (Figure 3b).

Abdominal cavity implantation: The dog was turned to a supine position. The lower abdominal region was shaved and disinfected. Incisions were made parallel to (and ~2 cm from) the groins to expose the abdominal muscles, which were then blunt-separated to expose the peritoneum. The peritoneum was opened. Eight scaffolds (four/side) were placed into the abdominal cavity and sutured to the parietal peritoneum to ensure a direct contact with the parietal peritoneum and omentum. Finally, the wound was closed in layers (Figure 3c).

After the operations, all animals were given daily intramuscular injections of penicillin (80 × 10^4^ IU) for 3 days. The animals were sacrificed by injecting an overdose of sodium pentobarbital after 1 month, 3 months and 6 months. The grafts were harvested and the tissue around the grafts was carefully removed. The general morphology of the specimens were observed.

### 4.3. Sample Preparation

Samples were fixed in 4% paraformaldehyde for 2 weeks at room temperature, rinsed with deionized water, dehydrated in an ethanol series and embedded with polymethyl methacrylate (PMMA) as per the method of our previous publications [35]. Then the samples were sectioned using a slicer (Microm HM360E, Thermo Fisher Scientific, Waltham, MA, USA) to yield 8 μm thick slices.

### 4.4. Histology, Histomorphometry, and Compression Tests

The slices were treated with H&E staining. The new bone formation, vascularization, inflammatory response and material–tissue interactions in the grafts were observed with optical microscopy (Nikon TS-100, Yokohama, Kanagawa, Japan). The percentage of the new bone area and the density of blood vessels were utilized to reveal the effects of the size and shape of the macro-pore structure on the osteoinduction and vascularization of the scaffolds. Vascular density: Each slice was randomly selected from 400 magnified fields of view, and the number of small vessels with a diameter less than 20 μm in each field of view was counted and averaged to represent the number of blood vessels per 0.2 mm^2^ unit area.

Compression tests (INSTRON 5567 Q4052, Canton, MA, USA) were performed to investigate the mechanical improvement of the scaffolds after the implantation. Referencing the ASTM D1621–2016 and ISO 844 standard test methods [56], the mechanical properties of the scaffolds were evaluated under the compression test at a strain rate of 0.5 mm/min using a 1 N load measurement and 0.005 mm deformation measurement accuracy. Four specimens were used for each scaffold.

### 4.5. Mathematical Models of the Macroporous Structure of 3D Scaffolds and Microfluidic Pathway

#### 4.5.1. Geometry Reconstruction

A μ-CT scanner (μCT-50, SCANCO Medical AG, Bassersdorf, Switzerland) was used to acquire images of both scaffolds with a voxel resolution of 20 μm. The threshold gray value was determined by matching the material volume obtained from the Archimedes measurements to that of the solid 3D volume calculated from the images in the evaluation (SCANCO Medical AG, Bassersdorf, Switzerland).

#### 4.5.2. CFD Model Creation

Our preliminary study found that two types of scaffolds (the HASAs and HAPPs) had complementary macro-porous structures [17]. The reconstruction images of the HASAs showed that the spheres accumulating type tends to the hexagonal close-accumulation (Figure 1a). Therefore, this study selected the hexagonal close-accumulation mode for the HASAs and its three-dimensional inversion mode for the HAPPs (Figure 1e), to construct two types of calculation models using CAD software SolideWorks 2014 (Dassault Systems S.A, Waltham, MA, USA). According to the macro-pore size and macro-pore shape of the actual scaffolds, the macro-pore sizes or sphere sizes of each model were set to 0.4 mm and 1.5 mm, respectively, and the sizes of the connective pores were set to 0.16 mm and 0.6 mm (the values were set according to the actual ratio of the connective pore size/macro-pore size = 0.4), respectively. Four models simulating the actual scaffold were used for the subsequent finite element analysis.

The fluid was defined as blood (viscosity of 0.033 Pa∙s, density of 1050 kg/m^3^, and the velocity-inlet of 0.01 m/s) according to previous studies [25,46], which was steady, 3D, laminar, and had a fully developed flow all over the scaffold. The Navier-Stokes equations governing fluid flow were solved by finite element software, Fluent 6.3 (ANSYS, Canonsburg, PA, USA). A no-slip boundary condition was applied on the flow channel wall. The flow speed near the walls was very low. The pressure was free at the flow channel outlets. To ensure the accuracy of flow simulation, each model was processed, in duplicate, four times.

#### 4.5.3. Mesh and Numerical Simulation

With an irregular geometry no analytical solution is known, so shear stress must be estimated from the grids adjacent to the wall with the finite element method. Meshing was implemented in the geometry of the pore structure of the scaffold, and the elements of the model were obtained. The flow channel space was broken into a large number of elements, each comprised a set of cubic grids.

The model simulated a Newtonian fluid, and the Navier-Stokes equations describing the flow inside the pore structure of the scaffolds were solved. After the solution was completed, the postprocessing was undergone for quantifying the wall shear rates, as well as the velocity profiles, along the flow channels.

In order to reflect the variation of the flow fields in the different macro-pore structures, the center cross section of the area where the fluid flowed through the macro-pore and the interconnected pore in CFD models was selected as the flow field analysis contrast section.

### 4.6. Statistical Analysis

The results were expressed as mean ± standard deviation. The differences between the two groups were analyzed with a t-test. The differences between the three groups were analyzed with an analysis of variance (ANOVA; SPSS 15.0, IBM, Armonk, NY, USA) and a Tukey multiple comparison test. A *p*-value < 0.05 was considered statistically significant.

## 5. Conclusions

In this study, the correlation between the size and shape of macro-pores and the osteoinduction of HA porous scaffolds was systematically studied. An animal model of ectopic implantation was used to explore the relationship between the macroscopic pore structure characteristics of the scaffolds and the microfluidic dynamic environment, which was further confirmed by a finite element simulation. The results showed that the macroscopic pore structure is a key factor determining the microfluidic dynamic environment of the scaffold, which in turn affects the bone formation and distribution of the scaffold. The dynamic environment of a microfluid acts as an important intermediate pathway, connecting the macrostructural properties and regulating the osteoinduction of these materials. The results of this study have provided experiment and theoretical bases toward quantitative analysis research of the correlation between the microfluid dynamic environment and the ectopic osteogenesis of calcium phosphate ceramics, and the optimal fabrication of calcium phosphate ceramics with controlled osteoinduction. It should be noted that the scaffold pore model used for the finite element analysis in this study is a scaffold model with ideal structural features, rather than a 3D reconstruction model of the actual scaffold. This caused some discrepancy between the computational simulation results and the actual experimental results. However, this difference does not affect the final conclusion of this study.

## Figures and Tables

**Table 1:** Histological summary of ectopic bone formations of HA porous scaffolds implanted in dorsal muscle and abdominal cavity.

Implant Time	Scaffold	Dorsal Muscle			Abdominal Cavity		
		New Bone Location	New Bone Formation (%)	Vessel Density (mm^−2^)	New Bone Location	New Bone Formation (%)	Vessel Density (mm^−2^)
1 M	L-HASAs	\	\	2.34 ± 0.55	\	\	1.86 ± 0.31
	S-HASAs	\	\	1.60 ± 0.79	\	\	1.43 ± 1.02
	L-HAPPs	Central	4.27 ± 1.85	2.67 ± 0.79	Central	3.06 ± 0.94	1.83 ± 0.23
	S-HAPPs	\	\	1.64 ± 0.21	\	\	1.39 ± 0.83
3 M	L-HASAs	Outer	10.36 ± 1.48	3.18 ± 0.37	Outer	5.47 ± 1.93	2.59 ± 0.41
	S-HASAs	Outer	6.18 ± 2.5	3.31 ± 0.72	Outer	1.74 ± 0.47	2.13 ± 0.47
	L-HAPPs	All	16.57 ± 2.79	4.12 ± 0.83	All	6.38 ± 2.05	3.15 ± 0.37
	S-HAPPs	Central	2.14 ± 1.33	2.58 ± 0.62	Central	1.28 ± 0.34	1.75 ± 0.15
6 M	L-HASAs	All	22.27 ± 2.89	4.02 ± 0.46	Outer	10.28 ± 2.06	3.17 ± 0.51
	S-HASAs	Outer	14.0 ± 3.67	4.78 ± 0.45	Outer	5.65 ± 1.17	2.87 ± 0.39
	L-HAPPs	All	28.31 ± 3.06	6.44 ± 0.75	All	15.93 ± 3.87	4.38 ± 0.53
	S-HAPPs	All	7.61 ± 2.48	3.11 ± 1.03	Central	3.94 ± 1.33	1.90 ± 0.63

## Data Availability

Not applicable.
